# Contactless Size Reference in Forensic Photography—Design and Verification of the Novel FreeRef-1 System

**DOI:** 10.3390/s23083790

**Published:** 2023-04-07

**Authors:** Luuk A. H. Schiks, Maura Cook, Laurentius R. Lipman, Arjan P. van Dijke, Kim Hutchinson, Paul van den Hoven, Arjo J. Loeve

**Affiliations:** 1 Department of Biomechanical Engineering, Faculty of Mechanical, Maritime and Materials Engineering, Delft University of Technology, Room F-0-200, Mekelweg 2, 2628 CD Delft, The Netherlands; 2 University of Amsterdam, Master Forensic Science, Science Park 904, 1098 XH Amsterdam, The Netherlands; 3 Team Crime Scene Investigations at Division Biological Traces & Division Digital and Biometric Traces, Netherlands Forensic Institute, Laan van Ypenburg 6, 2490 AA The Hague, The Netherlands; 4 Co van Ledden-Hulsebosch Center for Forensic Science and Medicine, 1098 XH Amsterdam, The Netherlands

**Keywords:** measurements, crime scene investigations, forensic research, forensic photography

## Abstract

In photographs of evidence in forensic investigations, physical size references (e.g., rulers or stickers) are often placed next to a trace to allow us to take measurements from photos. However, this is laborious and introduces contamination risks. The FreeRef-1 system is a contactless size reference system that allows us to take forensic photographs without having to be close to the evidence, and allows photographing under large angles without losing accuracy. The FreeRef-1 system performance was assessed using technical verification tests, inter-observer checks and user tests with forensic professionals. The results show that the measurements taken with photos using the FreeRef-1 system were at least as accurate as those taken using conventional techniques. Furthermore, with the FreeRef-1 system, even photographs taken under strongly oblique angles provided accurate measurements. The results suggest that the FreeRef-1 system will facilitate photographing evidence even in hard-to-reach places, such as under tables and on walls and ceilings, while increasing the accuracy and speed.

## 1. Introduction

**Figure 1:**
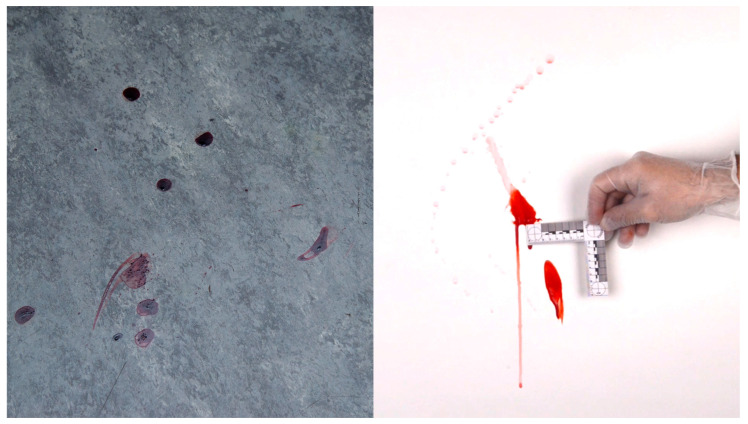
(**Left**) Blood stains on a floor, photographed without a size reference, making its dimensions impossible to judge. (**Right**) Blood stain on a white wall, with a physical size reference held close to it by an assistant, which introduces a risk of touching and altering or transferring the trace material.

Forensic photography is essential for and is a major part of crime and accident scene investigations, as well as in many evidence investigation and documentation steps in forensic labs. It is used for collecting and registering relevant information, for maintaining a chain of evidence, and for transferring all kinds of information to parties in the criminal justice system [1,2,3]. It is often hard, or even impossible, to tell a scale from a photograph, such as with a blood stain on a plastered wall, or a shoeprint on a plain floor (Figure 1 left). Therefore, it is common practice to place a physical size reference, such as a ruler or reference sticker, next to details, objects or traces of which the size might be relevant [4,5]. With the use of such references, the real-world dimensions of objects can be determined from just a photograph.

Although the use of size references is often an absolute necessity, there are several drawbacks to having to place them physically [2,4,5]. An investigator will have to stand close to the evidence, often having to step over other traces or objects, which takes time and introduces risks of disturbing the scene. Any rulers may subsequently be placed at different locations, requiring the rulers to be cleaned before each move to avoid contamination. When photographing traces on vertical surfaces (e.g., on walls) or the underside of a surface (e.g., on ceilings or under tables), one may have to first climb up to a trace to place reference stickers, work in difficult poses, or may have to ask a colleague to hold up the size reference while the photo is being taken (Figure 1 right).

Eliminating the need for physical size references would achieve the following:

- Reduce risks of evidence disturbance and contamination;
- Reduce the time needed for investigating crime or accident scenes and for processing evidence in the lab;
- Reduce the required manpower for investigating crime or accident scenes;
- Potentially reduce the financial cost of evidence processing and crime or accident scene investigations.

Once a photo has been taken with a physical size reference, performing any measurements on the photo requires several manual user actions, which may be prone to errors. The dimensions of the size reference must be determined (in pixels for digital photos and in mm for hard copies) to obtain a scaling factor that can be used to translate the dimensions of the object or trace of interest in the photo to its real-world dimensions. This process is rather laborious and measurement accuracy is highly sensitive to perspective effects and to deviations from orthogonal viewpoints [3,4,5]. Therefore, investigators often struggle at crime scenes to find a pose or position from which an item and the size reference can be orthogonally photographed. This introduces further delays, scene disturbance and contamination risks.

In order to facilitate, increase the safety of, and speed up placing and using size references in evidence photography, an automated size reference projection and processing system was developed. The system, named “FreeRef-1”, was developed by the authors in collaboration between a technical university and a national forensic institute (see authors’ affiliations), and with support from the national police of the same country. In order for this system to be suitable for forensic use, it should, besides complying to usability and safety requirements, above all provide a robust and reliable size reference. Therefore, the aim of the current work was to present the FreeRef-1 system and to assess its measurement accuracy in a broad range of situations relevant in forensic practice at crime or accident scenes and in laboratories.

## 2. Materials and Methods

### 2.1. The FreeRef-1 System

**Figure 2:**
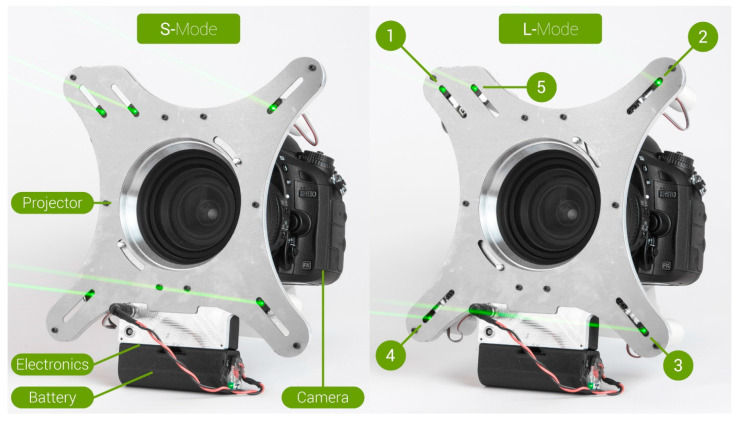
The FreeRef-1 projector mounted on a Nikon D610 camera with an 18–135 mm objective; however, this is not the objective used in the verification tests, which is shown in Figure 4. (**Left**) Lasers in S-mode, closest to the objective. (**Right**) Lasers in L-mode, furthest from the objective. Lasers 1 to 4 are the size reference lasers. Laser 5 is the mode laser that is used to detect the selected mode in the photos.

The FreeRef-1 system consists of (1) a projector and (2) custom-made stand-alone software. The **projector** (Figure 2) contains five 1 mW green lasers that project their beams parallel with the camera lens at known center distances. The lasers project four dots into a square pattern that has a constant size at any distance from the camera. Each laser is situated in a fixture with calibration screws that enable fine adjustments of that laser’s projection angle. The projector contains a mechanism that allows moving the lasers closer to the lens (Small or S-mode) or further from the lens (Large or L-mode) to enable users to select the size of the projected dots pattern to fit around the item to be photographed. Lasers 1 to 4 are used for the size reference. The fifth laser is a “mode-laser” that is used by the FreeRef-1 software to automatically detect in a photograph whether the projector was used in S- or L-mode. This is deduced from the relative position of the mode-laser dot with respect to the four reference dots, which changes depending on the used S- or L-mode. The projector can be mounted on any common camera lens with a filter thread on its front element by using any commonly available Nikon SY-1 adapter ring or any other brand step-up ring from the filter thread diameter to any convenient diameter Nikon SY-1 adapter, to which the projector is affixed. In the future, the projector will also be suitable for use with a mobile phone by using a 3D-printed adapter.

**Figure 3:**
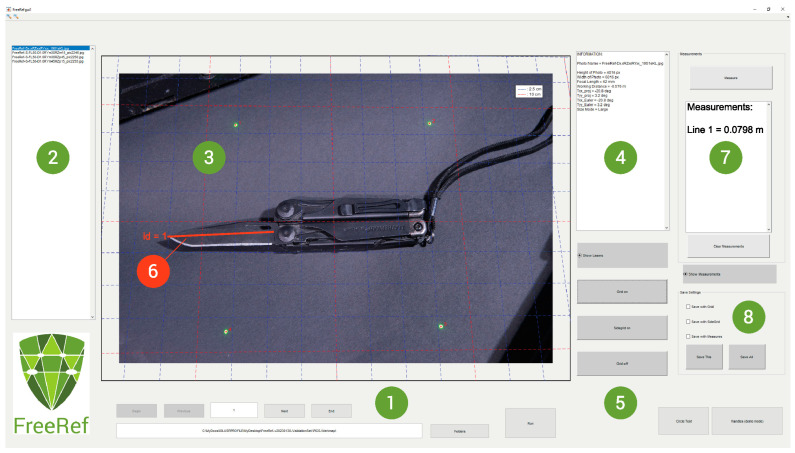
The FreeRef-1 (version 20230130) software interface. (1) Folder and file selection, and analysis run button. (2) List with photos found and analyzed in selected folder. (3) Viewing and working area showing the photo selected in area 2. The locations of the detected laser dots are marked with stars and a size reference grid is placed as an overlay, oriented with the same rotation as the calculated projection plane rotations. (4) Summary of photo info and detection results. (5) Buttons for hiding/displaying grid around or on the photo, and laser detection points on or off. (6) Measurement performed by the user in the shown photo. (7) Overview of measurement numbers and real-world lengths on the projection plane. (8) Picture-saving options selection buttons for exporting annotated files with grid or measurements added.

The FreeRef-1 **software** was developed in Matlab (version R2019b, Matwhorks Natick, MA, USA). The user can load a folder with JPG photo files taken with the projector switched on, which is then automatically processed by the software to detect the laser dots (Figure 3). If the software cannot find an appropriate set of dots, it will prompt the user to indicate them. If any laser dots are detected incorrectly, the user can override the results. The software combines EXIF information about the camera sensor, camera objective focal length and about the FreeRef-1 projector with the locations of the detected laser dots. This is used to first calculate the horizontal and vertical rotations of the plane on which the laser dots are visible in the photo (the projection plane), with respect to the photo camera sensor. With these rotations known, a transfer function from pixel-space dimensions in the 2D photograph to real-world 3D locations in meters on the projection plane is determined. This way, measurements can be taken anywhere on the virtual plane through the projected laser dots, while taking perspective effects into account.

In the software user interface (Figure 3), users can choose to show or hide the detected locations of the laser dots and a grid showing real-world dimensions on or around the photograph. The interface also allows users to perform measurements on the photo with just two mouse clicks, which automatically draws and labels the measured distance. This produces annotated photos in which the user determines what is or is not shown. Once finished annotating, the user can export the annotated photos as JPG files to a folder of their choosing. These exported files retain their original file names, but are extended with a standard, software-determined indication of the export settings. This way, original files can never be accidentally overwritten and will never be changed by the FreeRef-1 software, making it safe to use for applications in which a chain of evidence is important.

### 2.2. Technical Verification and Sensitivity Tests

Because of the goniometric calculations underlying the conversion from pixel-space dimensions to real-world dimensions with the FreeRef-1 system, it was expected that inaccuracies in determining the laser dots’ locations in 3D would increase with the following factors:

- **Working distance**, which is the distance between the camera and laser dot projection plane (at larger distances, the dot pattern becomes smaller in the photograph and small deviations in detecting the laser centers give relatively large dimensional errors).
- **Focal length** of the camera lens (with wider view angles, the dot pattern also becomes smaller in the photograph).
- **Projection plane angles** (with larger angles, small angle deviations cause large deviations in how far from the camera a dot is calculated to be).

When the detected locations of the laser dots deviate from their real-world locations, the calculated **resulting** projection plane angles **Rxx** and **Ryy** (degrees), as well as the resulting real-world dimensions calculations, will also be incorrect. Therefore, a test was designed to determine how accurate the calculated real-world dimensions are when using the FreeRef-1 software for measurements in photos under a broad range of use conditions.

#### 2.2.1. Test Setup

**Figure 4:**
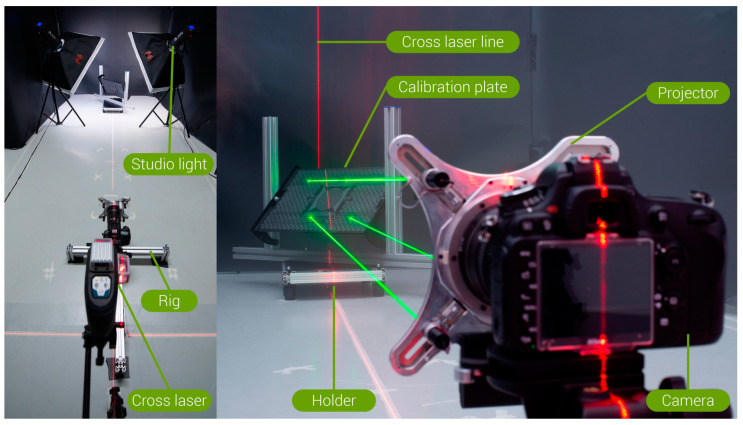
Test setup used to capture the photos for the verification tests: (**Left**) as viewed from behind and above the camera towards the calibration plate and (**Right**) as viewed from the left, behind and leveled with the camera.

**Figure 5:**
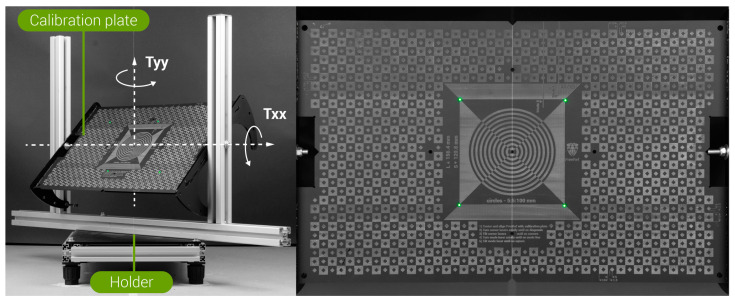
Calibration plate used for the verification tests. (**Left**) The calibration plate in its holder, with the horizontal rotation axis Txx and vertical rotation axis Tyy indicated. (**Right**) Frontal view of the calibration plate.

**Figure 6:**
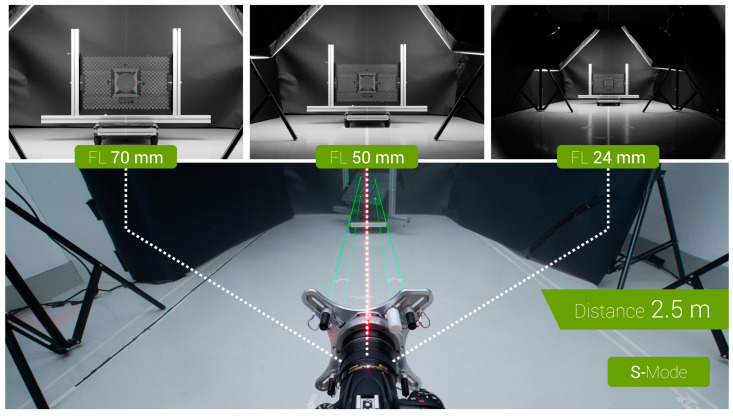
Test setup used for the verification tests, with three example verification pictures at the 2.5 m working distance in the S-mode, showing the effect of using different focal lengths.

The FreeRef-1 projector was mounted (Figure 4) on a Nikon 24–70 mm f/2.8 objective (ser.nr. 326137, Nikon, Shinjuku, Tokyo, Japan) on a Nikon D610 body (ser.nr. 6105711), set at the sensor sensitivity ISO800, aperture f/8 and 1/20 s shutter speed, and image quality setting RAW+JPG High (resolution 6016 × 4016 pixels). On the zoom ring of the camera lens, the 50 mm focal length position was marked for easy repeatable setting. The photo camera was mounted on a rig with a precision tripod head (Manfrotto 410). Using a self-leveling cross laser (Bosch GLL 3-80 C 3 601 K63 R00, 0.3 mm/m accuracy) and laser distance measure (Bosch GLM40 3 601 K72 900, 1.5 mm accuracy), the projector was leveled and pointed orthogonally at a vertically leveled, custom-made calibration plate (Figure 4, Figure 5 and Figure 6) by orienting the tripod head accordingly. The calibration plate functioned as the projection plane. Its center was aligned with the camera lens axis. The calibration plate was situated on a moveable holder. (Figure 4 and Figure 5). This holder had adjustable supports for leveling its base, and allowed rotation of the calibration plate around a horizontal axis (**true angle Txx**) and a vertical axis (**true angle Tyy**) in steps of 15° in each direction from the neutral pose: 0° rotation around both axes, being when the plate was normal to the camera objective optical axis. The rotation axes intersected in the calibration plate center to ensure that the working distance was independent of Txx and Tyy (Figure 5).

Around the setup, a black paper screen was put up to create an even background for optimal performance when detecting the dots. This did not influence the system’s calculation or measurement accuracy in any way, but did help to avoid false positive laser dot detections and resulted in cleaner photographs. Two studio LED lights (FalconEyes LPS-1000TD, set at 100% brightness and 4550K color temperature) with soft boxes were placed on tripods on both sides of the calibration plate to provide even, soft lighting, balanced with the camera settings.

The calibration plate (Figure 5) had laser-engraved markings that indicated where the laser dots should be when the projector was in the S- and where in the L-mode, which guided the calibration of the projector’s reference lasers’ orientations. In addition, circles and squares with known dimensions were engraved on the calibration plate to enable the assessment of the FreeRef-1 software measurement accuracy by measuring these known distances in the FreeRef-1 software.

The projector and the test setup were calibrated and aligned in both the S-mode and the L-mode with the lasers turned on. With the calibration plate and the projector leveled and orthogonal, the orientation of each reference laser in the projector was set, using its calibration screws, to have each laser dot hit the correct target on the calibration plate.

#### 2.2.2. Photo Acquisition

The calibration plate with the laser dots projected onto it was photographed with the camera on which the projector was mounted. All photos were shot with the FreeRef-1 projector first in L-mode on one day and later in S-mode on another day. This was performed at various working distances, focal lengths and projection plane angles. Working distances of 1.0, 0.5, 2.5 and 5.0 m were used in that order for pragmatic reasons. At each working distance, rotation Txx around the horizontal axis was varied from 45° to +45° in steps of 15°. At each Txx, rotation Tyy around the vertical axis was varied from 45° to +45° degrees in steps of 15°.

At each Tyy, a photograph was shot at different focal lengths from the set of 24, 50 and 70 mm (Figure 6). Within each Txx, all 50 mm focal length photos were grouped and taken one after another by varying Tyy, while keeping the focal length constant to ensure that the focal length within this group was always identical. The 24 and 70 mm focal length shots were taken together for each Tyy in a separate group in the same Txx, because these focal lengths were at the ends of the camera lens zoom ring rotation and could, hence, be reliably repeated. This way the focal lengths were kept as consistent as possible, while minimizing the number of times Txx and Tyy had to be set.

At the 0.5 m working distance, only the 24 and 50 mm focal lengths were used, as with the 70 mm focal length, the image was too zoomed in to capture the laser dots. At the 5.0 m working distance, only the 50 and 70 mm focal lengths were used, as with the 24 mm focal length, the laser dots were too small to be reliably distinguished from this large distance. In the S-mode, the 5.0 m working distance was skipped altogether, as this mode was unlikely to be useful in forensic practice at this distance. Tables with the detailed orders of testing are provided in [6].

**Figure 7:**
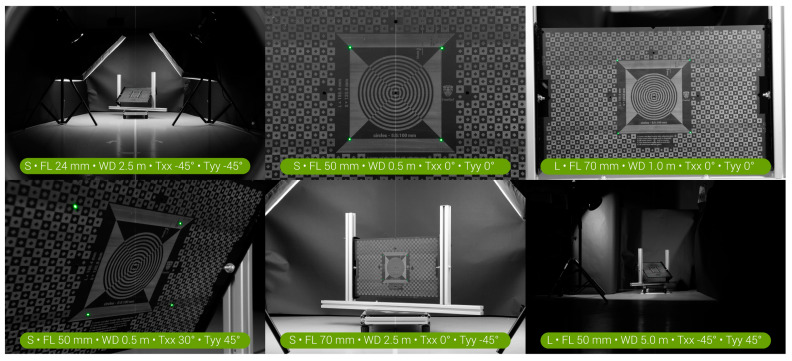
Six arbitrary examples of the photos with the calibration plate used in the verification tests for the tested use conditions. S = S-mode, L = L-mode, FL = focal length, WD = working distance.

Figure 7 shows an arbitrary selection of the resulting photos used in the verification tests for the tested use conditions. The complete set of photos taken in all use conditions is provided in an online data repository [6]. This dataset also contains two short illustrative stop-motion movies (S-mode and L-mode) that show all photos sequentially (for quick viewing, these can be viewed on YouTube via tinyurl.com/FreeRef-S-VerSeq-2023 and tinyurl.com/FreeRef-L-VerSeq-2023).

#### 2.2.3. Data Preparation and Processing

**Photo pre-processing** was performed for all 490 photos taken in L-mode and all 392 photos taken in S-mode in Adobe Camera Raw by desaturating all color channels except for green, the laser color. This was conducted to minimize the risk of obtaining false positive dot detections. False positive detections would slow down the analysis, as these had to be manually corrected while loading the photos. Desaturation did not affect the measurement accuracy, as the locations of the dots remained unaltered.

**The projection plane angles** calculated by the FreeRef-1 software were checked by running all photos through the software (version 20230130). For each photo, the **resulting projection plane angles Rxx** and **Ryy** (around the horizontal and vertical axis, respectively, see Figure 5) calculated by the software were registered and compared to the known angles Txx and Tyy, as set during the tests. **The absolute angle errors Exx** and **Eyy** (for rotations around the horizontal and vertical axis, respectively) were defined by subtracting the true angle from the FreeRef-calculated angle and taking the absolute value of this difference. The angle errors were used to check for abnormalities in the data or to find the sources of any large real-world dimensions measurement errors. Small angle errors were not interpreted as an expression of angle detection accuracy, because the calibration plate holder was such that any set angle Txx or Tyy could easily be a degree off from the intended value due to clearances in the rotation mechanism.

**Figure 8:**
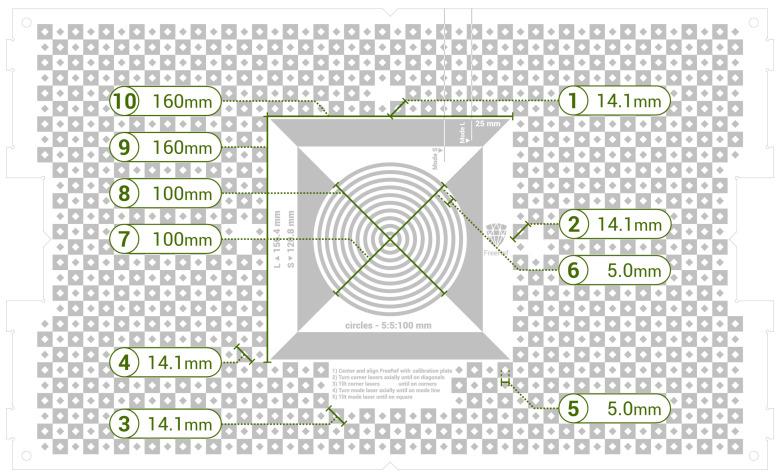
Scheme of the calibration plate (in color negative, because the black parts in the image are laser-engraved on the plate, which turns white where it is engraved) and the ten verification lines used for the verification tests.

**Verification of the real-world dimensions** calculated by the software was achieved by conducting in-photo measurements in the FreeRef-1 software by two hired assistants who underwent a one-hour work instruction and practice round and then independently took measurements using either the S- or the L-mode photographs. For every photo, the lengths of the 10 verification lines shown in Figure 8 were measured using the FreeRef-1 software’s measure function by clicking the two endpoints of each verification line. This was done while maximizing the FreeRef-1 software window on a 15.6” computer monitor (HP Zbook Studio 16). In some photographs, verification line 4 was outside the image frame for long focal lengths combined with close working distances and large Txx and Tyy angles; in those cases, the diagonal of another similar square as close as possible to the intended one, but still in-frame, was used. In about 20 photos per mode, the laser dots were detected on the wrong place due to highlights in the pictures or due to user errors, which resulted in wrong calculations of the projection plane rotation angles and wrong measurements. These faults were only discovered during data pre-processing and were resolved by sending those images back to the assistants and asking them redo the measurements after taking care of proper detection of the laser dots.

The resulting software-calculated dimensions were compared to the known actual lengths of these lines. This was achieved by calculating absolute percentage length measurement errors for each FreeRef-measured length. These were calculated by taking the absolute difference between the measured and actual length, expressed as a percentage of the actual length. This resulting measure of accuracy included not only the accuracy of the software, but also any potential deviations introduced by the assistants who had to accurately click the correct measurement points.

**Inter-assistant variation** was checked by having the two assistants repeat all in-photo measurements of the 10 verification lines in an arbitrarily selected subset of 21 of the photos taken in L-mode for the assistant who initially processed the S-mode photos and vice versa for the other assistant. The difference between these 210 inter-user measurements per mode and their paired 210 verification measurements was used as an estimate of the part of the real-world dimensions’ calculation error not caused by the software, but by the assistants’ targeting inaccuracy.

**Statistical analysis** was conducted in RStudio (RStudio, PBC, v2021.09.0 Build 351). To investigate what factors most affect the FreeRef-1 system’s performance, the measurement data were checked for effects of the independent variables (working distance, focal length, and the two projection plane angles Txx and Tyy) on the dependent variable (absolute percentage measurement error). There were too many degrees of freedom to allow using an n-way MANOVA for analyzing the effects of all the combined factors, while taking the ten verification line measurement errors as separate dependent variables. Therefore, for each photograph, the verification line measurement errors were taken as a single dependent variable. This was achieved for the S-mode and L-mode photographs separately.

Inter-assistant differences were checked using a two-tailed paired t-test to check for statistically significant differences (*p* < 0.05) between the two assistants’ measurements. Inter-assistant agreement was checked by creating Bland–Altman plots, comparing all the inter-assistant measurements to the same measurements taken by the other assistant in the main process, both for the measurement values in mm, as well as for the percentage absolute measurement error.

### 2.3. User Tests for Measurement-Procedure Validation

User tests were conducted to compare the accuracy of real-world sizes determined using the FreeRef-1 software to the accuracy achieved with conventional techniques using physical size references. It was expected that the measurements taken using the FreeRef-1 software would be non-inferior to, and less time-consuming than, those taken using conventional techniques. Unfortunately, it turned out that in the user tests (and only in these), an older version of the FreeRef-1 software was used that contained an error. Therefore, only the measurement data from the conventional techniques are reported here.

#### 2.3.1. Photo Acquisition

**Figure 9:**
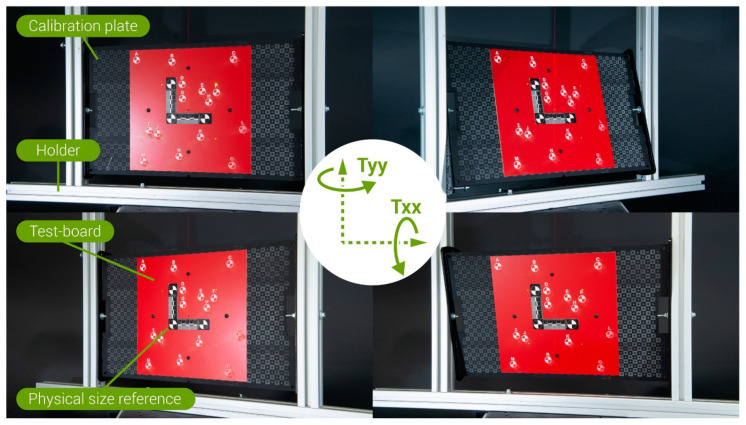
Four arbitrary examples of the S-mode photos with the test board used in the user tests.

To ensure that the ground truth values of any measurements by the test-users were unambiguous, the test-users were asked to measure distances between points indicated by target marks. A piece of 280 × 280 × 1.6 mm white and red dual-layered acrylic plate (article 2613030101 at www.kunststofshop.nl) was laser-engraved (using a Lion Lasers L4A-25W BabyLion Laser) with 15 targets marked with letters A through to O (Figure 9). The points were positioned on this test board such that a range of point distances between 3 and 30 cm was covered in vertical, horizontal and diagonal directions, spread across the full surface of the board. The actual point distances were measured between the centers of the target marks using a Mitutoyo digital caliper for point distances < 15 cm and a ruler for point distances > 15 cm (since these were out of range for the caliper) and are included in [6]. To enable participants to determine the point distances using their own preferred current techniques, a physical size reference of a type regularly used at crime-scenes (Loci Forensics B.V., photomacrographic scale, L-shape, 8 cm) was glued to the center of the test board.

Photos of the test board were shot using the same setup as for those taken for the technical verification and sensitivity tests (see Section 2.2.2), except here, the laser-engraved test board was mounted to the center of the calibration plate (Figure 9). Photos were shot in S-mode at a working distance of 1 m, using a focal length of 50 mm. Rotation Txx around the horizontal axis varied from −30° to +30° in steps of 15°. At each Txx, rotation Tyy around the vertical axis varied from −30° to +30° in steps of 15°.

#### 2.3.2. Test Procedure and Participants

A total of fifteen participants were included in the experiment. Five participants were crime scene investigators as part of the Netherlands Police, nine were forensic specialists as part of the Netherlands Police, and one was a forensic expert at the Netherlands Forensic Institute. Ethical approval for conducting this study was granted by the human research ethics committee of the university at which the last author is affiliated (approval number 2371). All participants provided their written, informed consent before partaking in the test. The informed consent form and study information sheet provided to participants can be retrieved from [6].

Each participant was assigned a set of five photographs of the test board, from each of which they were asked to determine three real-life point distances using either first the FreeRef-1 software, and then three other point distances using their currently preferred method, or vice versa (in pre-randomized order). The time participants took to determine each point distance was recorded, starting from the moment they were told which distance to determine, until the moment they read out the measured value.

Each set of five photos was determined by randomly selecting a photo of the test board from each of the following categories: Txx = 0° and Tyy = 0°; Txx = ±15° (positive or negative) and Tyy = 0, or vice versa; Txx = ±30° and Tyy = 0, or vice versa; Txx = ±15° and Tyy = ±15°; Txx = ±30 and Tyy = ±15°, or vice versa. The order in which the photos were presented to participants was randomized, with a photo from each category occurring in each position at least once. For each photo, the three point distances to be determined were randomly selected as a combination of small, large or medium size (<10 cm, 10–20 cm, >20 cm), and horizontally, vertically or diagonally oriented. A complete overview of the details of the protocol per participant is provided in [6].

#### 2.3.3. Data Analysis

**Error calculations** were performed for all point distances determined by the participants during the tests. These differences with the actual point distances were computed by subtracting the true distances from the participant-determined point distances. The absolute (magnitude) values of these errors were expressed as a percentage of the corresponding true point distances, along with the median and 75% interval of the FreeRef-1 technical verification data for the subset, using Tyy and Txx angles between −30° and 30° for reference.

## 3. Results

**Figure 10:**
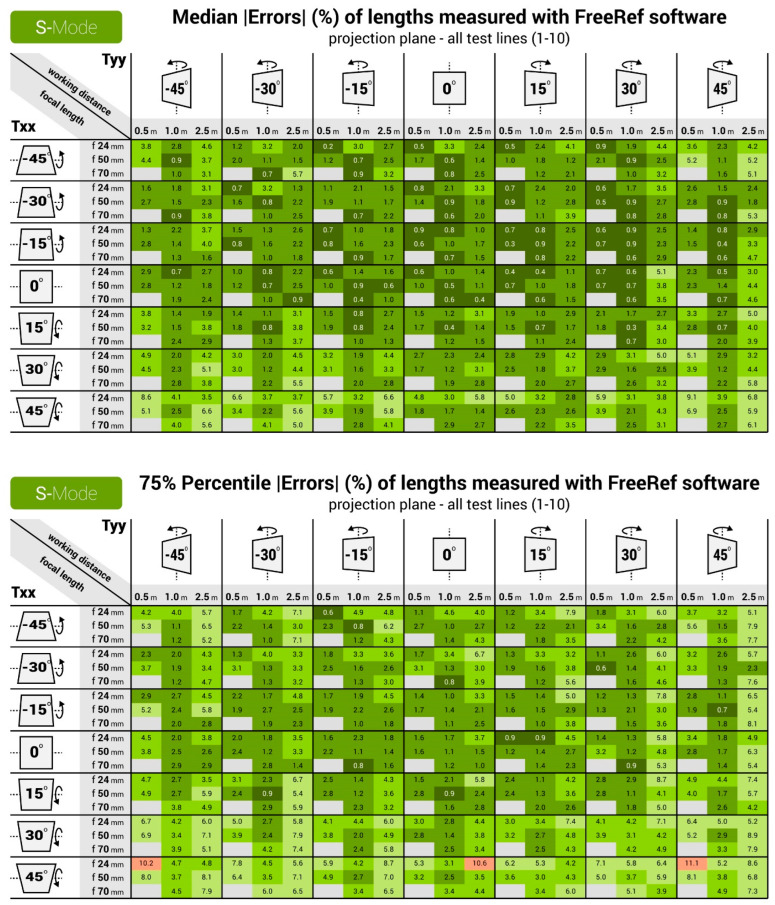
Heat maps for the **S-mode** verification measurements, showing the (**Top**) median and (**Bottom**) 75 percentile values of absolute percentage measurement errors for all tested focal lengths, working distances and calibration plate rotations Txx and Tyy.

**Figure 11:**
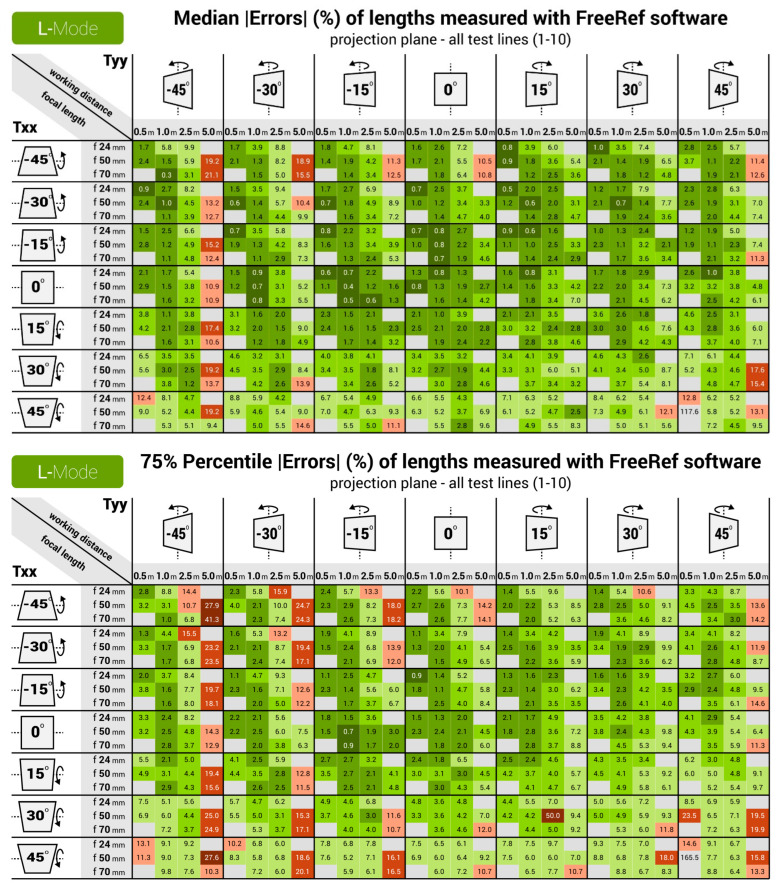
Heat maps for the **L-mode** verification measurements, showing the (**Top**) median and (**Bottom**) 75 percentile values of absolute percentage measurement errors for all tested focal lengths, working distances and calibration plate rotations Txx and Tyy.

Figure 10 and Figure 11 show the median and 75% quartile absolute percentage measurement errors for the S- and L-mode photographs. For the S-mode, all of the medians and the vast majority of the 75% quartile values are below the 5% absolute measurement error. The largest measurement errors were found for extreme values of Txx and Tyy, in particular when combined with a large working distance. For the L-mode, the accuracy turned out slightly lower than for the S-mode. It turned out that the FreeRef-1 projector L-mode calibration was slightly incorrect because the calibration plate holder was not fully leveled, as can be observed in the top right photo in Figure 7. As a result, the rotation angle detection performed slightly worse than for the S-mode photos. This underlines the necessity of designing a projector that is always properly mounted. In general, the L-mode still had median errors that were mostly under 5% for all situations, except for the 5.0 m working distance. At this distance, the perspective effects and sizes of the laser dots with respect to the image resolution were simply too small (see bottom right photo in Figure 7) to provide accurate rotation angle calculations at the largest tested rotations Txx and Tyy. There was one extreme outlier in the L-mode photos (focal length 50 mm, working distance 0.5 m, Txx and Tyy both 45°), with a median percentage absolute error of 117.6%. This was not due to the assistant errors or flaws in the measurement algorithm, but was caused by the software failing to detect the correct four laser dots. Due to this, Rxx and Ryy were incorrectly calculated, which affected all other outcomes. Although this data point could have been removed as an outlier, we decided to include it to illustrate the importance of correctly detecting the laser dots. All measurement data and the R-code used for analysis are provided in [6].

For the S- and the L-mode, the ANOVA test showed significant effects (all with *p* < 0.001) on the percentage absolute measurement error of the working distance, Txx and Tyy. For the L-mode, this was also the case for the focal length. Furthermore, there were interaction effects (all with *p* < 0.001) of working distance with Txx and with Tyy for the S- and L-mode, and of focal length with Tyy for the S-mode.

**Figure 12:**
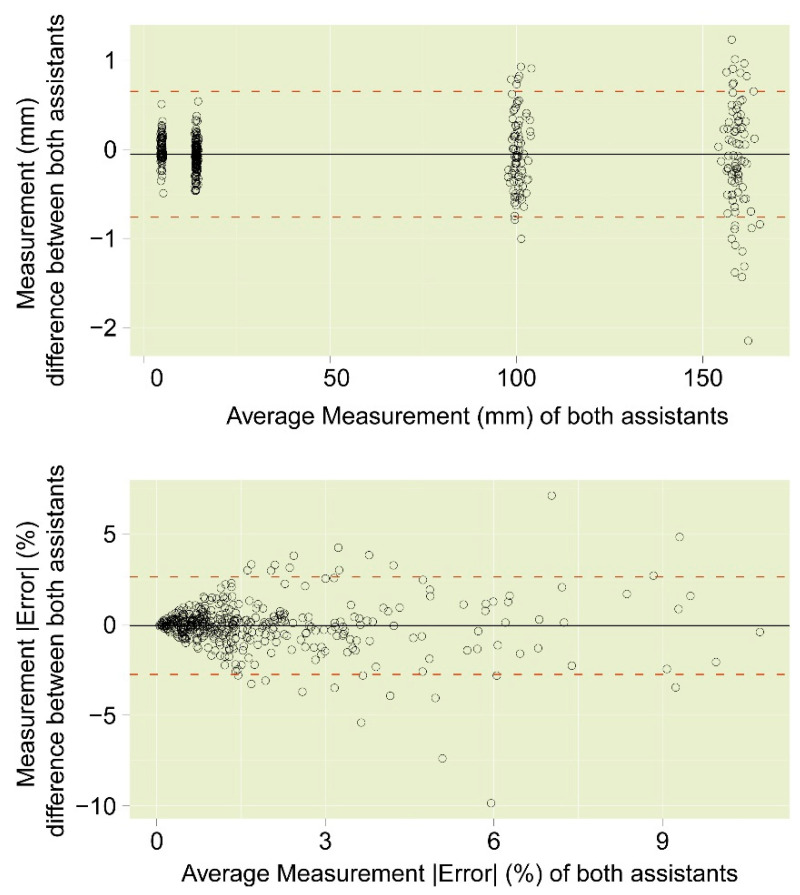
Bland–Altman plots of the inter-observer test results for (**Top**) the measurement values in mm and (**Bottom**) the percentage absolute measurement errors.

The inter-assistant t-test did not show any statistically significant differences between the two assistants (*p* = 0.32). The Bland–Altman plots (Figure 12) showed strong agreement between the two assistants. The largest absolute differences between the two assistants were between 1 and 2 mm, and were only found for the largest lengths of 160 mm. In terms of absolute percentage measurement errors, the inter-observer differences were in general not larger than about 2%.

### User Tests for Measurement-Procedure Validation

A total of 222 participant-determined point distances were included in the results, out of the originally intended 225. One participant refused to complete the three-dimension measurements using their currently preferred technique for one of their photos because they felt they could not do so accurately for the large angle the photo was taken at (15°/30°). There were four conventional techniques that were used by the participants as their go-to approach, which were as follows:

- Eleven out of fifteen participants performed the current techniques measurements by zooming in on the photograph until it was at the 1:1 scale on the screen using the ruler in the photograph as reference, and then measuring distances on-screen using a physical ruler.
- Two participants measured both the distances and reference ruler on-screen using a physical ruler and computed the real-life distances using the determined scaling factor.
- One participant used Adobe Photoshop and the reference ruler in the photograph to print the photos out at the 1:1 scale, and then used a physical ruler on the printed picture to determine the dimensions.
- One participant used Adobe Photoshop to determine the pixel length of the reference ruler in the photograph, computed the pixel/cm ratio and then converted the pixel measurements of the distances to real-life dimensions.

Only the two participants who used Adobe Photoshop in their process performed image corrections to correct photos taken at an angle with respect to the trace, before performing current technique measurements.

**Figure 13:**
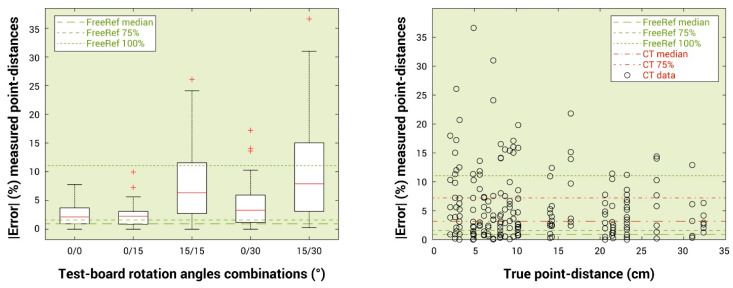
Point distance measurement errors made by the participants in the user tests. (**Left**) Box plot showing the participants’ measurement errors for the different combinations of Txx and Tyy rotations of the test board. Boxes indicate the 25 and 75% quartiles, the red center line in each box indicates the median and the whiskers are at the edges of the 0 and 100% quartiles, with red plus signs showing outliers. (**Right**) Scatter plot of the same data, sorted by true point distance. CT = current technique.

The absolute percentage errors in the point distances determined by the participants using conventional techniques are shown in a scatterplot in Figure 13. The median and 75% percentile of the FreeRef-1 technical verification errors were both below the median of the current technique errors. This was the case for all categories of projection angles included in the user tests, as can be observed in the boxplot in Figure 13. The average time it took participants using the FreeRef-1 software was 22.0 s (SD = 22 s). With conventional techniques, the average time was 48.4 s (SD = 88 s). For the conventional techniques, the first point distance participants determined from a given photograph took about five times longer than the succeeding two measurements (102, 23, and 20 s, respectively). This was because for the first measurement, a user must compute a scaling factor, zoom to scale, or print the photo.

## 4. Discussion

**Figure 14:**
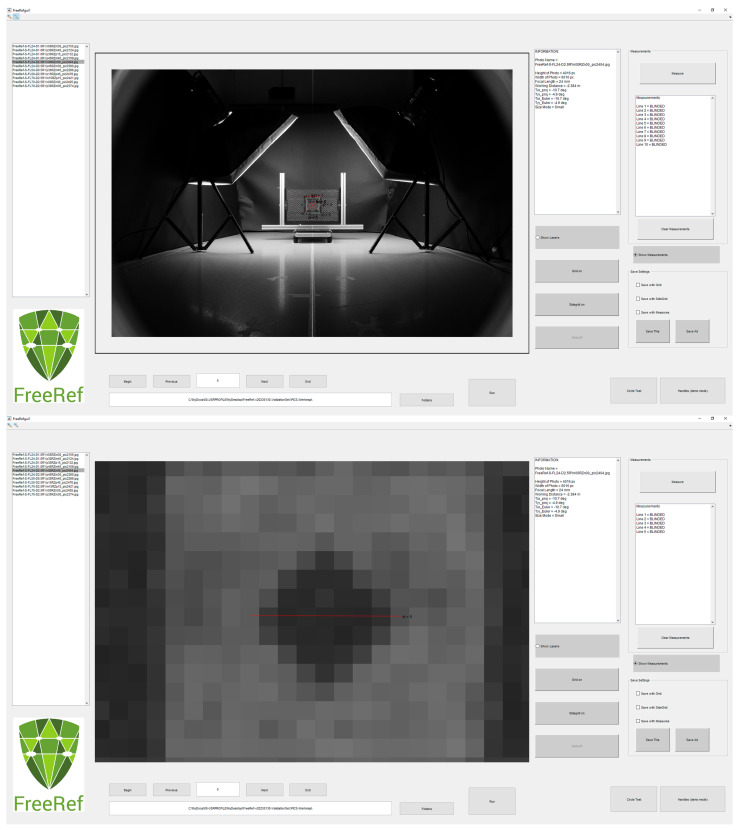
Example of how the measurement lines were placed in the FreeRef-1 software by the assistants. (**Top**) Interface with photo entirely zoomed out and all 10 verification line measurements shown. (**Bottom**) Interface with photo zoomed in to measure verification line number 5, showing the challenge of targeting the correct corners of the square.

The FreeRef-1 measurement errors are generally only a few percent, including the user error. From near to far and from straight to 45° dual-angled photographs, the FreeRef-1 accuracy was shown to be well within the limits of practical forensic use in lab conditions. Furthermore, the inter-assistant tests showed that inter-assistant variations amounted for measurement percentage errors of the same order of magnitude as the verification results in general. This suggests that the measurement errors mainly originate from user inaccuracies and not from inaccuracies in the system itself. Figure 14 shows an arbitrary example of how the measurement lines were placed in the FreeRef-1 software by the assistants. It is clear that at large working distances, one has to zoom in so much for the smallest verification lengths that accurately clicking on the intended points on the calibration becomes a matter of a well-aimed guess between pixels. Hence, the measurement errors at these large distances, and the statistically significant effects of working distance and of focal length in interaction with Tyy seem to be simply caused by the limits of photo resolution. Therefore, users should, in a future improved software version, be made aware, either through user instructions or FreeRef-1 software warning messages, when measurements taken in photographs are too close to the photo resolution limits.

The FreeRef-1 verification results matched and mostly even surpassed the accuracy reached by police forensic investigators using their currently preferred techniques. This also holds for photographs taken at large angles (beyond 15° to 30°) that are currently deemed unsuitable for in-photo measurements. In addition, despite the users having had no prior experience with the FreeRef-1 software, they did perform faster using the FreeRef-1 software than with their own preferred methods. Yet, it should be noted that the accuracy of the measurements taken by the participants using their currently preferred methods in this test should by no means be interpreted as being representative of size referencing accuracy in actual forensic case work. This is because in the experiment, the participants were asked to conduct measurements in strongly angled photographs, which they would normally not do. Furthermore, the used photographs were clean, well-lit and taken in a lab setting. Conditions in forensic practice will often be much more complex, e.g. because crime scenes are usually messier than labs. Nonetheless, the results show that the FreeRef-1 system can accurately measure even when the projection plane angles are large. Hence, the FreeRef-1 system will allow for reliable measurements in angled photos, and hence allows us to photograph traces in hard-to-reach places that could previously not, or only with much effort and inconvenience, be photographed with reliable size references.

LiDAR (light detection and ranging, or 3D laser scanning) [7] could be a potential alternative to physical size references, as it provides a contactless, remote measurement of real-world dimensions while taking photographs. However, LiDAR is a built-in technique. Broadly introducing this in the forensic field would require significant investments in replacing all the existing photo cameras and mobile phones currently being used for evidence photography, which would first need to be developed. Furthermore, next to requiring large amounts of data to store LiDAR data with a full set of photogrammetry images, its absolute accuracy for small objects > 10 cm is only about 1 cm (using an iPhone) [7], which is up to 10 times worse than the FreeRef-1 system, depending on the distance and projection plane angles. When using specialized LiDAR equipment, the accuracies of point locations can be in the sub-centimeter range, but besides being still insufficiently accurate for small dimensions, these devices are bulky and expensive and require expert operators [8,9,10].

Stereoscopic photogrammetry is another means for obtaining 3D real-world dimensions from sets of 2D photos. A test on artificial wounds on a mannequin resulted in measurement errors of about 6.1% on average with stereoscopy, against 13.95% when using conventional techniques (measuring in photographs, similar to our user study) [11]. However, stereoscopic photogrammetry requires dual-objective or dual-camera systems, which are generally expensive and require well-trained operators [11]. Monoscopic photogrammetry using a mobile phone camera and open-source software could make photogrammetry cheaper and more widely available [12]. However, these techniques still always require having a physical size reference in at least a part of the photographs, and require a set of convergent photographs that are taken under approximately the same lighting conditions. Hence, the user must move around the object of interest many times to capture a plurality of photographs from various angles and ensure proper and constant light conditions for image stitching. The former is undesirable when considering contamination and disturbance of the scene and both are often not possible at a crime scene.

In contrast to the potential alternatives discussed above, the FreeRef-1 projector is both a simple and highly accurate tool, potentially suitable for use with any kind of camera, including mobile phones, without the need to adapt the camera itself. The FreeRef-1 contactless, projection-angle-tolerant, accurate measurements potentially offer great advantages in the forensic field. For example, even with a wide-angle photograph taken at an extreme angle between the projection plane and the camera, one could strongly zoom in on a blood spatter pattern and be able to measure, for example, blood stains of a millimeter in size with an error below 0.05 mm. Due to the measurement errors being relative and not absolute by nature, this new technique enables us to take a single shot of an entire wall with blood spatter patterns on a crime scene and still be able to measure the individual stains within a few percent. Because the photograph does not have to be taken at an as straight as possible angle, as is essential in the current techniques, one could easily photograph evidence under tables, on ceilings above objects and various other hard-to-reach places and still have reliable size references.

Because the FreeRef-1 provides contactless placement of size references and accurate measurements at large Txx and Tyy angles up to at least 2.5 m working distances, the system is believed to be very suitable for use in traffic accident investigations, damage investigations of ship bows (currently often requires photographing from another boat on moving water), placing a real-world size reference in 3D scans, and measuring crop growth in greeneries (currently a laborious manual process). The future market-ready FreeRef project is aimed to cost about the same as a regular external camera flash, that is, about EUR 500. In return, the contactless, projection-angle-tolerant, accurate FreeRef-1 system will offer many opportunities for saving time, workforce and money, while increasing measurement accuracies and reducing contamination risks due to the reduced need for photographers and assistants to be close to the evidence. This is particularly valuable because size references are often placed for precautionary reasons in case measurements are needed in the future, but regularly end up not being used at all.

One of the limitations in this study is the potential inaccuracy of establishing the ground truth of verification line lengths and point distances. If the ground truth deviated from the actual lengths or distances, this may have also caused the measurement errors to be slightly affected. However, because the errors were averaged per ten, this is unlikely to have had any relevant effect on the outcomes. The calibration plate rotations Txx and Tyy may have also slightly deviated from their intended positions; therefore, the reported values may have been for rotations that were not exactly the Txx and Tyy values reported. Yet, the FreeRef-1 detected projection plane rotations Rxx and Ryy were mostly within 1° and almost always within 2° of Txx and Tyy, except for the 2.5 m working distance. At the 2.5 m working distance, the detected Ryy was still usually within 2°, sporadically up to 5° and eight times up to 10 or 20° due to wrong laser detections, likely caused by the laser dots being too difficult to discern, due to photo resolution limitations. At the 5 m working distance, the accuracy should be considered too low for practical use, although it is expected that with better alignment of the projector on the camera and improved laser dot detection algorithms, this can be further improved. Overall, the results suggest that the FreeRef-1 system accurately detects the projection plane rotation angles and that the test setup was fit for purpose.

Improvements of the FreeRef-1 projector are being implemented to make it robust and light-weight. In addition,, the FreeRef-1 software may benefit from machine learning algorithms to improve the speed, user experience, and accuracy of the laser dots detection in practical situations. The system should be tested with crime scene investigators on mock-up crime and accident scenes, which are often much messier environments than our lab, and in forensic laboratories to assess its practical usability. Finally, the usability, cost effectiveness and performance of the final system and its workflow from crime scenes to use in court should be compared to the current techniques before its implementation in forensic practice.

## 5. Conclusions

The FreeRef-1 system is a novel solution for placing size references in evidence photographs. The current study shows that the system enables fast and accurate measurements of real-world dimensions in photographs taken even under extreme rotations of 45° over two axes, with respect to the photographed plane. Until recently, with conventional techniques, photographs taken under such angles were generally deemed unsuitable for measurements in forenthe sic contexts. FreeRef-1 enables us to take accurate measurements of even mm sized traces in overview photos, and will speed up and facilitate taking photographs of hard-to-reach places at crime scenes without needing an assistant. Consequently, FreeRef-1 may contribute to preventing professionals from accidentally damaging or contaminating forensic evidence on a crime scene, while improving measurement accuracy and speed compared to conventional techniques.

## Data Availability

The data presented in this study are openly available in the 4TU data repository at DOI 10.4121/22180438.
